# A biotin-guided hydrogen sulfide fluorescent probe and its application in living cell imaging†

**DOI:** 10.1039/d0ra06524b

**Published:** 2020-10-01

**Authors:** Chen Zhang, Jiewen Zhang, Zhiqiang Xu, Kun Zang, Feng Liu, Jun Yin, Ying Tan, Yuyang Jiang

**Affiliations:** State Key Laboratory of Chemical Oncogenomics, Key Laboratory of Chemical Biology, Tsinghua Shenzhen International Graduate School, Tsinghua University Shenzhen 518055 P. R. China tan.ying@il.ccnu.edu.cn; Key Laboratory of Pesticide and Chemical Biology, Ministry of Education, College of Chemistry, Central China Normal University Wuhan 430079 China yinj@mail.ccnu.edu.cn; School of Pharmaceutical Sciences, Tsinghua University Beijing 100084 P. R. China

## Abstract

Hydrogen sulfide (H_2_S), a well-known signaling molecule, exerts significant regulatory effects on the cardiovascular and nervous systems. Therefore, monitoring the metabolism of H_2_S offers a potential mechanism to detect various diseases. In addition, biotin is significantly used as a targeting group to detect cancer cells exclusively. In this work, a biotin-guided benzoxadizole-based fluorescent probe, NP-biotin, was developed for H_2_S detection and evaluated in normal liver cell (LO2) and liver cancer cell (HepG2) lines. Results reveal that NP-biotin can detect cellular H_2_S with high sensitivity and selectivity. Moreover, NP-biotin has been confirmed to possess the ability to target cancer cells under the guidance of the biotin group.

## Abstract 2

A biotin-guided hydrogen sulfide fluorescent probe has been shown clearly to possess the ability to target cancer cells.

## Graphical abstract

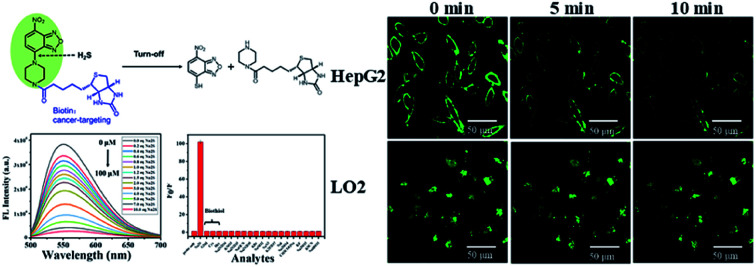

## Introduction

Like carbon monoxide (CO) and nitric oxide (NO), hydrogen sulfide (H_2_S) is well known as a gaseous mediator. H_2_S regulates the cardiovascular system^1–5^ and nervous systems^6,7^ and also exerts anti-inflammatory effects.^8,9^ So far, many studies have suggested that endogenous H_2_S is mainly produced from cysteine by CBS (cystathionine β-synthase) or CSE (cystathionine γ-lyase) enzymes, which are responsible for the synthesis of H_2_S *in vivo*.^10–12^ In addition, several pathways for H_2_S synthesis have been reported,^13,14^ in which the enzymatic actions of CBS and CSE on cysteine have been regarded as the predominant driving force.

As a signaling molecule, various concentrations (from nM to μM) of H_2_S are found in different tissues and biological fluids,^15^ thus the excess generation or paucity of H_2_S indicate disease status. In recent years, it has been reported that dysregulation of H_2_S metabolism is related to neurodegenerative diseases, such as Parkinson's, Alzheimer's, and Huntington's diseases.^16^ However, research on the physiological and pathological functions of H_2_S is still in its preliminary stage compared the extensive studies on CO and NO. Monitoring the production and distribution of cellular H_2_S could help to understand how it stimulates biological response and interacts with signaling pathways, further illustrating its relationship with diseases.

Compared with the traditional methods, using the fluorescence imaging technique to detect cellular H_2_S has numerous advantages. Several benefits include monitoring the production of H_2_S in real-time and displaying the spatial distribution of H_2_S without destructive sampling. Besides, the fluorescence imaging has high sensitivity and selectivity towards its target based on fluorescent probes.^17–24^ To assist the fluorescence imaging of H_2_S, it is necessary to develop a fluorescent probe with excellent performance. Based on reports of the good selectivity of the piperazinyl-NBD-based probe towards H_2_S, we selected piperazinyl-NBD as a response group for H_2_S in designing our probe.^25^

Many cancer cells often overexpress vitamin receptors (such as folate and biotin) on the surface of the cell membrane to aid the transduction of signals and uptake of nutrients, which could promote the fast growth and proliferation of cancer cells.^26,27^ Based on the specific recognition between vitamins and their corresponding receptors, vitamins are commonly used in a drug delivery system to target cancer cells exclusively.^28–30^ In previous reports, researchers confirmed that the biotin-modified probes would be more selectively taken up by biotin-positive cancer cells than by biotin-negative cells.^31–34^ Taking this into consideration, the biotin group was introduced into our probe to increase its cancer-targeting ability.

In this work, a biotin-guided piperazinyl-NBD-based fluorescent probe NP-biotin for H_2_S is reported. Biotin was selected as a cancer-targeting group, and piperazinyl-NBD was used as a response group for H_2_S. NP-biotin exhibited great sensitivity and selectivity for H_2_S. In the living cell imaging, NP-biotin successfully detected cellular H_2_S and targeted the cancer cells *via* the binding with biotin receptors under guidance of the biotin group.

## Experimental section

### Materials and instruments

All reagents are obtained commercially. UV-Vis spectra were obtained from spectrometer (Beckman DU 800, USA) and fluorescence spectra were measured on a fluorescence spectrophotometer (SPEX Flurolog 3-TCSPC instrument, USA). ^1^H and ^13^C NMR spectra were recorded on nuclear magnetic resonance spectrometer (Bruker AVIII-400, Germany) and mass spectra were recorded on mass spectrometry (AB Sciex QSTAR Elite, USA). Water was prepared by the Milli-Q purification system.

### Synthesis of NP-biotin

**Scheme 1:**
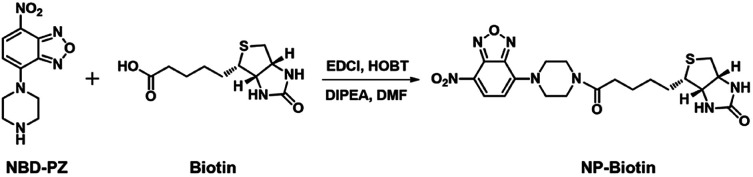
The synthesis of NP-biotin.

The synthesis procedure of our probe, NP-biotin, is shown in Scheme 1. Under argon atmosphere, NBD-PZ (50 mg, 0.20 mmol) was added into a mixture of biotin (68 mg, 0.20 mmol), DIPEA (0.10 mL), EDCI (58 mg, 0.30 mmol), and HOBt (68 mg, 0.50 mmol) in DMF (5.0 mL). The mixture was stirred overnight at room temperature. After the addition of 20 mL water, the mixture was extracted with ethyl acetate and washed three times by water, then dried with Na_2_SO_4_. After the solvent was removed, the crude product was purified by silica column chromatography with dichloromethane/methanol (20 : 1) to obtain the final NP-biotin product, which displayed an orange color, with a yield of 45%. The structure of NP-biotin was confirmed by ^1^H and ^13^C NMR spectrum (Fig. S1 and S2†). ^1^H NMR (400 MHz, DMSO-d_6_): *δ* (ppm) = 8.53 (d, *J* = 8 Hz, 1H, Ar-H), 6.62 (d, *J* = 8 Hz, 1H, Ar-H), 6.47 (s, 1H), 6.39 (s, 1H), 4.31 (t, *J* = 8 Hz, 1H), 4.24–4.14 (m, 4H), 4.09 (s, 1H), 3.80–3.73 (m, 4H), 3.14–3.09 (m, 1H), 2.85–2.80 (m, 1H), 2.59 (d, *J* = 12 Hz, 1H), 2.38 (t, *J* = 8 Hz, 2H), 1.64–1.47 (m, 4H), 1.37 (t, *J* = 4 Hz, 2H). ^13^C NMR (101 MHz, DMSO-d_6_): *δ* (ppm) = 171.0, 162.6, 145.4, 144.7, 136.2, 121.0, 103.0, 61.0, 59.1, 55.4, 48.9, 48.5, 43.5, 32.0, 28.2, 28.0, 24.5. ESI-HRMS: *m*/*z* calcd for C_20_H_26_N_7_O_5_S [M + H]^+^ 476.1716; found: 476.1712.

### Spectroscopic measurements

The stock solution of NP-biotin (1 mM, DMSO) was prepared then stored in the dark until use. The stock solution of Na_2_S (the source of H_2_S) and other analytes (10 mM) were dissolved in water then diluted by PBS (10 mM, pH = 7.4) when used. For the spectroscopic measurements, the stock solution of NP-biotin was diluted to work solution (10 μM) by PBS (10 mM, pH = 7.4, 1% DMSO). In the titration experiment, Na_2_S was added step-wise into the NP-biotin work solution to observe the behavior of NP-biotin towards different concentrations of Na_2_S. Subsequently, the selectivity of NP-biotin was investigated by addition of the various analytes (100 μM) mentioned above. Also, the availability of NP-biotin was tested in solutions of different pH values, ranging 3.0–12.0. The kinetics between NP-biotin and Na_2_S (100 μM) were explored by recording the fluorescence change of NP-biotin at 550 nm upon the addition of Na_2_S. The fluorescence spectrum of NP-biotin was recorded under the excitation at 480 nm, and the slit width of the excitation and emission was set to 5 nm.

### Cell culture and living cell imaging

HepG2 and LO2 cells were cultured in Dulbecco's modified Eagle's medium supplemented with 10% fetal bovine serum in an atmosphere with 5% CO_2_ at 37 °C. Cells were seeded in glass-bottom culture dishes until attached and then incubated with the 10 μM probe followed by washing with PBS before imaging on a laser scanning confocal microscope (Olympus FV-1000-IX81). Emission was collected at the green channel (500–600 nm, excitation at 488 nm).

### Cytotoxicity assay

Cells were seeded in a 96-well cell culture dish (1000–10 000 cells per well). After cell attachment, the cell culture medium containing the probe from 0–100 μM was added for 24 h incubation. Then, the medium containing probe was replaced with the medium containing 10% CCK-8 reagent. After another 4 h of incubation, the absorbance of each well at 450 nm was measured with the microplate reader (TECAN Infinite Series M1000 Pro).

## Results and discussion

### Optical response of NP-biotin to H_2_S

**Fig. 1:**
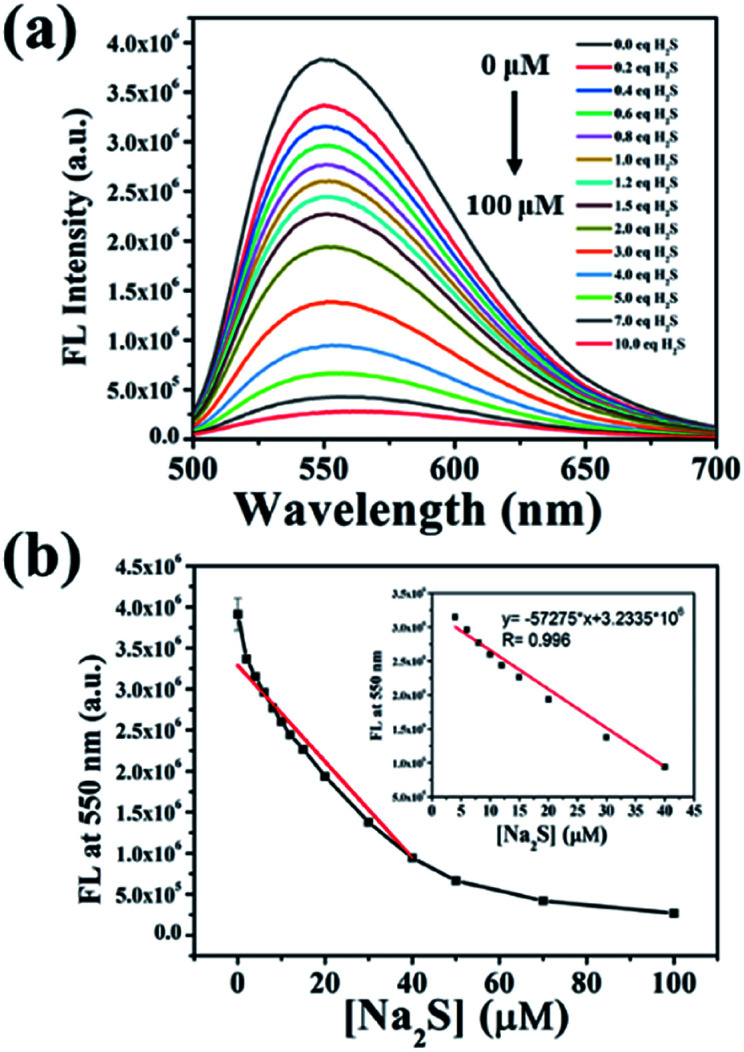
(a) Fluorescence spectra of 10 μM NP-biotin upon the addition of 0–100 μM under the excitation of 480 nm; (b) the relationship between fluorescence intensity of NP-biotin at 550 nm and Na_2_S concentration in the range of 0–100 μM (inset: linearity between the fluorescence intensity of NP-biotin at 550 nm and Na_2_S concentration in the range of 0–40 μM).

To explore the response of NP-biotin towards different equivalents of Na_2_S, we performed a titration experiment. The fluorescence spectra of NP-biotin were recorded after the Na_2_S stepwise addition. As shown in Fig. 1a, NP-biotin shows strong fluorescence emission at 550 nm, and the emission peak gradually decreased as the concentration of Na_2_S increased. A good linear relationship is seen between the fluorescence intensity at 550 nm and Na_2_S concentration in the range of 0–40 μM (Fig. 1b). Based on the 3*σ*/*s* principle,^35^ the limit of detection was calculated to be 3.69 nM, which is much lower than the physiological concentration of H_2_S (10–100 μM).^36–38^ NP-biotin exhibited great sensitivity for Na_2_S, which indicates its good potential in living cell imaging.

**Fig. 2:**
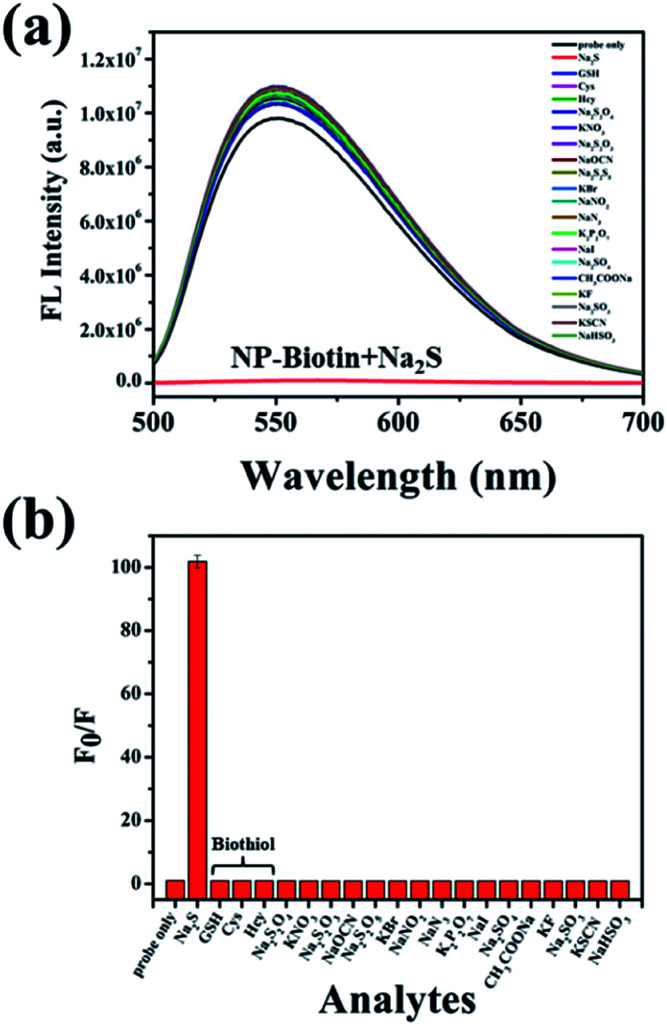
Fluorescence spectra (a) and *F*_0_/*F* at 550 nm (b) of 10 μM NP-biotin with various species (Na_2_S, GSH, Cys, Hcy, Na_2_S_2_O_4_, KNO_3_, Na_2_S_2_O_3_, NaOCN, Na_2_S_2_O_5_, KBr, NaNO_2_, NaN_3_, K_2_P_2_O_7_, NaI, Na_2_SO_4_, CH_3_COONa, KF, Na_2_SO_3_, KSCN and NaHSO_3_) under excitation at 480 nm (*F*_0_ represents the fluorescence intensity of NP-biotin and *F* represents the fluorescence intensity of NP-biotin in the presence of other guests respectively).

The selectivity was evaluated by Na_2_S and other analytes, including biothiols GSH, Cys, and Hcy. As shown in Fig. 2a, only Na_2_S could efficiently quench the fluorescence of the probe, and the fluorescence intensity at 550 nm was reduced about 100-fold after the addition of Na_2_S (Fig. 2b). It is worth mentioning that GSH, Cys, and Hcy had almost no effect on the fluorescence performance of NP-biotin. Furthermore, the response of NP-biotin to Na_2_S was also explored in the presence of other analytes (Fig. S3†). The results show that the existence of other species do not affect the quenching of NP-biotin by Na_2_S. It is also interesting to note that the probe showed excellent selectivity towards Na_2_S, which implies it could be applied in complex biological systems.

**Fig. 3:**
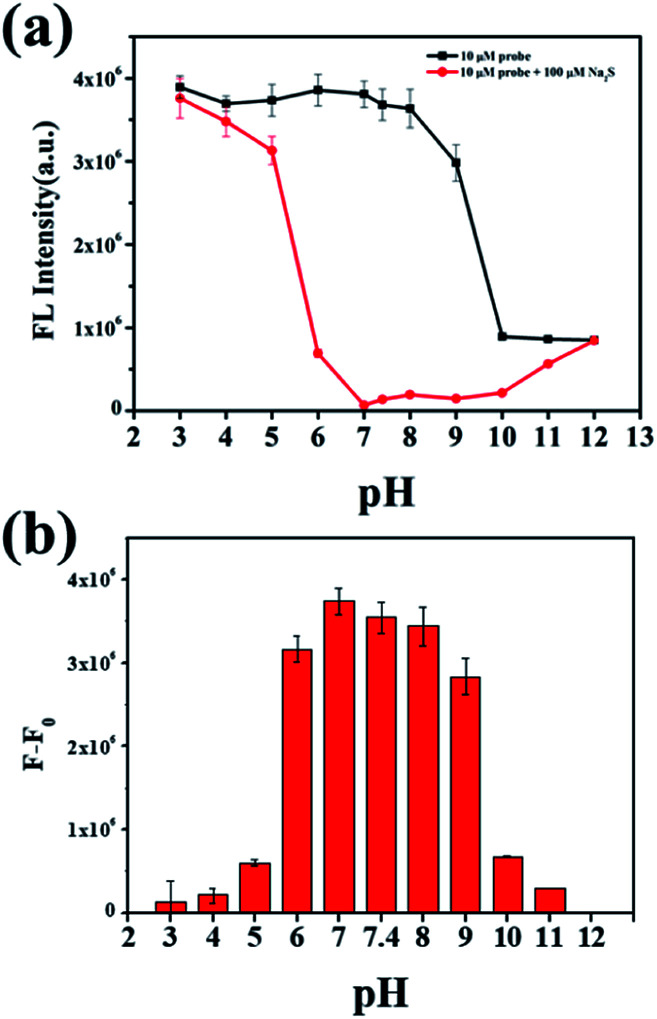
(a) Fluorescence intensity at 550 nm of 10 μM NP-biotin with or without Na_2_S (100 μM) under different pH conditions; (b) the fluorescence change of 10 μM NP-biotin under different pH conditions (*F*_0_ represents the fluorescence intensity of NP-biotin, and *F* represents the fluorescence intensity of NP-biotin with Na_2_S).

To investigate the behavior of NP-biotin with or without Na_2_S at different pH values, a series of phosphate buffers with different pH values were prepared. The difference between the fluorescence intensity at 550 nm in the absence (black line in Fig. 3a) and presence (red line in Fig. 3a) of Na_2_S could reflect the availability of NP-biotin towards Na_2_S under different pH conditions. In Fig. 3b, a dramatic reduction of fluorescence intensity at 550 nm occurred in the pH range from 6.0–9.0 and reached a maximum at pH 7.0, which is closed to the physiological pH of humans. Impressively, these results indicate that NP-biotin works well in physiological pH conditions.

**Fig. 4:**
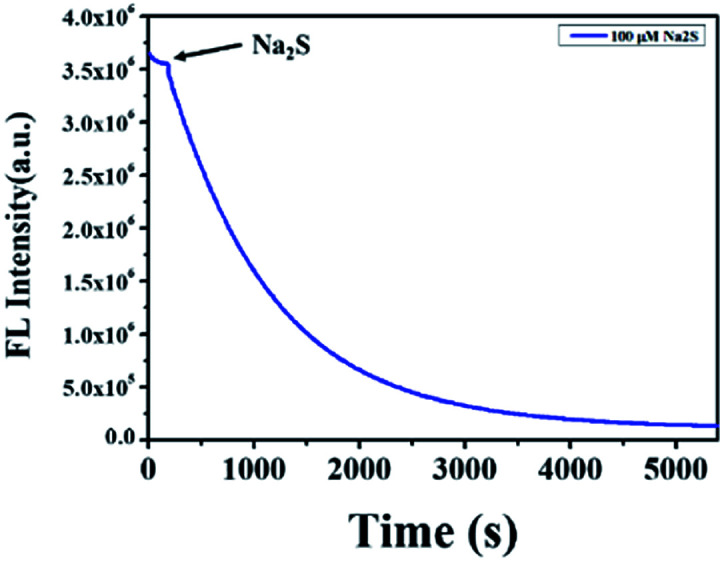
The time-dependent fluorescence intensity at 550 nm of 10 μM NP-biotin upon reaction with Na_2_S (100 μM).

To explore the kinetics between NP-biotin and Na_2_S, the time-dependent fluorescence intensity of NP-biotin at 550 nm was monitored after the addition of Na_2_S. As shown in Fig. 4, the fluorescence intensity of NP-biotin decreased significantly as soon as the Na_2_S was added and reached a minimum after about 1 h. The pseudo-first-order rate was calculated to be 7.4 × 10^−3^ S^−1^ by fitting the fluorescence intensity with a single exponential function. These data imply the possibility of NP-biotin to detect H_2_S in a real-time manner.

**Fig. 5:**
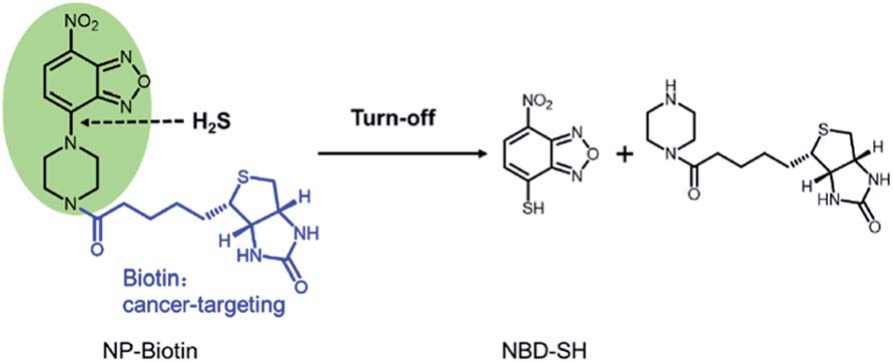
The proposed mechanism for the reaction of NP-biotin with H_2_S.

In order to verify the mechanism of the reaction between the probe and Na_2_S, the methanol solution before and after the reaction was analyzed by mass spectrometry. The sensing mechanism was explored by ESI-HRMS. As shown in Fig. S4 and S5,† [NP-biotin + H]^+^ (*m*/*z*: 476.1712, calcd for C_20_H_26_N_7_O_5_S ([M + H]^+^: 476.1716)) was detected in the mass spectrum of the probe solution before Na_2_S was added. [NBD-SH-H]^−^ (*m*/*z*: 195.9821, calcd for C_6_H_2_N_3_O_3_S ([M − H]^−^: 195.9820)) was detected in the mass spectrum of the probe solution after Na_2_S was added. These data confirm that in the proposed sensing mechanism (Fig. 5) based on the nucleophilicity, H_2_S cleaves the C–N bond between piperazinyl and benzoxadizole of NP-biotin to ultimately produce NBD-SH.

### Fluorescence imaging of NP-biotin in living cells

In the following cell imaging experiments, HepG2,^39–41^ a liver cancer cell line, was used as biotin receptor-positive cell, and a normal liver cell line LO2 (ref. 42) was selected as biotin receptor-negative cell.

To explore the application of NP-biotin in the detection of intracellular hydrogen sulfide, we examined the effects of probes at different concentrations on cell viability using the CCK-8 Kit. HepG2 cells were treated by the probe with different concentrations for 24 h. The cytotoxicity results show that the cell viability of HepG2 had almost no change with 0–50 μM probe treatment and remained about 80% with the 100 μM probe, which indicates that the probe exhibits low cytotoxicity (Fig. S6†). Hence, we selected a concentration of 10 μM for subsequent cell imaging experiments.

**Fig. 6:**
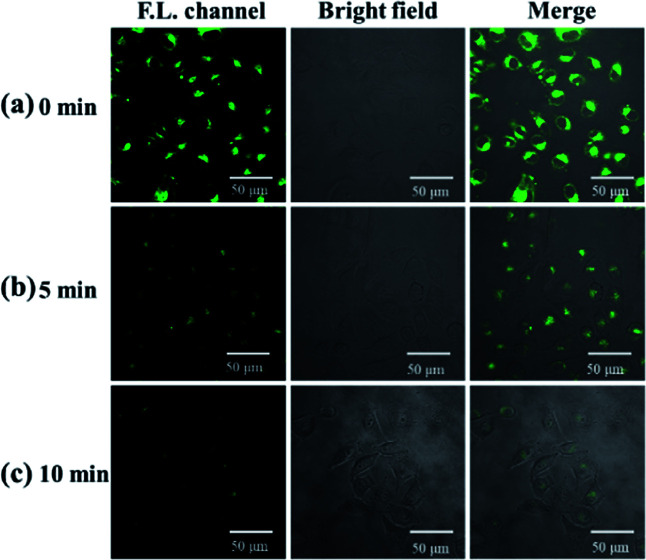
Fluorescent images of LO2 cells treated with 10 μM NP-biotin for 1 h (a) 0 min after exogenous hydrogen sulfide (50 μM Na_2_S) were added; (b) 5 min after exogenous hydrogen sulfide were added; (c) 10 min after exogenous hydrogen sulfide were added.

To verify the ability of probe molecules to detect exogenous hydrogen sulfide, LO2 cells were selected as the target cells. After incubation with 10 μM NP-biotin for 1 h, strong fluorescence was observed inside LO2 cells (Fig. 6a). After 50 μM Na_2_S was added, the fluorescence gradually decreased and almost turned off 10 min (Fig. 6c). These results demonstrate that the probe could detect exogenous hydrogen sulfide.

**Fig. 7:**
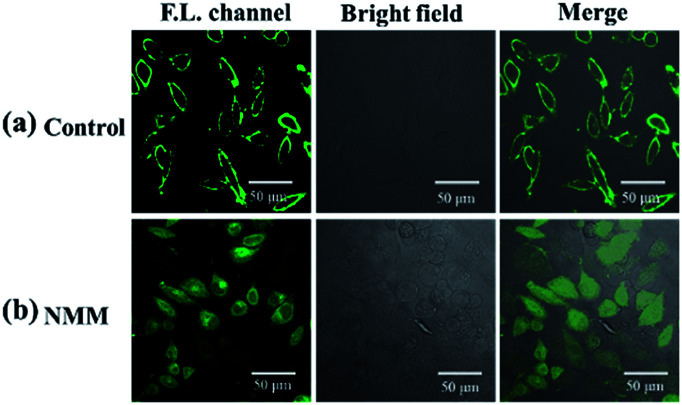
(a) Fluorescent images of HepG2 cells incubated with 10 μM NP-biotin for 1 h; (b) fluorescent images of HepG2 cells pretreated with 1 mM NMM for 1 h then incubated with 10 μM probe for 1 h.

Comparatively, in fluorescent imaging of HepG2, fluorescence was observed on the cell membrane but almost no fluorescence could be observed in the cytoplasm (Fig. 7a). It has been reported that cancer cells usually express excessive hydrogen sulfide compared to normal cells;^43,44^ thus, the weak fluorescence inside the HepG2 cells may be caused by the high concentration of hydrogen sulfide. NMM (*N*-methylmaleimide), a hydrogen sulfide scavenger, was tested to verify our speculation. HepG2 was pretreated with 1 mM NMM, which was used to consume endogenous H_2_S before incubation with the probe. The fluorescence appeared when the cells were pretreated with NMM, as shown in Fig. 7b. At the moment, the pH of endosome and lysosome in HepG2 cells was acidic, but the significant fluorescence of the NP-biotin was shown in the cells. So this experiment can confirm that the fluorescence of the NP-biotin would not be significantly reduced by being exposed to acidic pH in endosome and lysosome. From what has been discussed above these results indicate that the weak fluorescence was caused by the high concentration of H_2_S in HepG2.

**Fig. 8:**
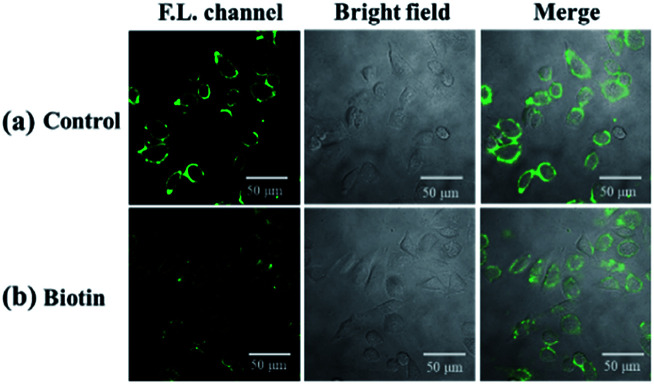
(a) Fluorescent images of HepG2 cells incubated with 10 μM NP-biotin for 1 h; (b) fluorescent images of HepG2 cells incubated with 2 mM biotin and 10 μM NP-biotin for 1 h simultaneously.

**Fig. 9:**
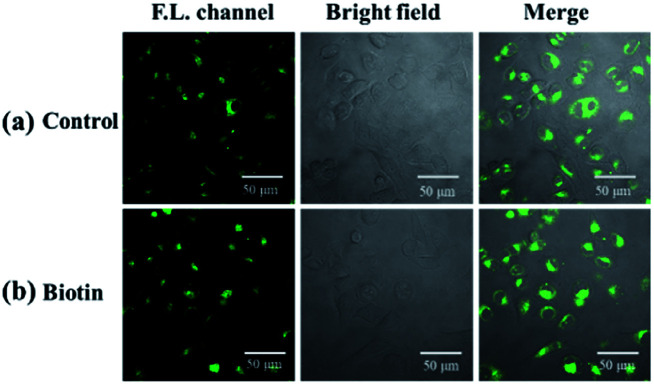
(a) Fluorescent images of LO2 cells incubated with 10 μM NP-biotin for 1 h; (b) fluorescent images of LO2 cells incubated with 2 mM biotin and 10 μM NP-biotin for 1 h simultaneously.

Cell imaging was further used to investigate the tumor targeting ability of the probe. In this assay, both biotin receptor-positive cells (HepG2) and biotin receptor-negative cells (LO2) were selected as subjects. It is suggested that the bright fluorescence on the cell membrane of HepG2 may be attributed to the biotin receptors, which could recognize and bind to the biotin group of our probe.^45–47^ To confirm this, biotin (final concentration of 2 mM) was added with probe, in which biotin was used as competition for biotin receptors on the cell membrane with the probe. As shown in Fig. 8, the presence of biotin significantly reduced the fluorescence intensity on the cell membrane of HepG2, which indicates that biotin occupied a certain amount of biotin receptors and limited the sites available for NP-biotin. This phenomenon demonstrates the targeting ability of NP-biotin towards cancer cells *via* biotin receptors. As expected, the co-incubation of biotin with the probe had no influence on LO2 cells, a biotin receptor negative cell line (Fig. 9). These results prove that NP-biotin possesses a cancer-targeting function by binding to the biotin receptor.

**Fig. 10:**
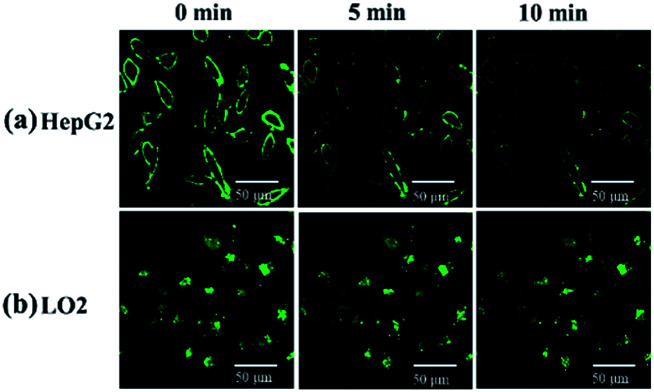
(a) HepG2 and (b) LO2 were imaged every 5 min after incubation with 10 μM NP-biotin for 1 h and washing by PBS.

After incubation with 10 μM probe for 1 h and washing by PBS, HepG2 and LO2 cells were observed every 5 min by the microscope. The fluorescence on the cell membrane of HepG2 gradually decreased over time, which suggests that the probe was transported from the membrane to cytoplasm and then recognized by the H_2_S inside HepG2 cells (Fig. 10a). On the contrary, the fluorescence of LO2 cells did not decrease (Fig. 10b), indicating that the probe in the cytoplasm was not completely quenched. This result demonstrates that HepG2 cells exhibit a higher H_2_S concentration than LO2 cells, which agrees with the previous studies that reported cancer cells usually express more H_2_S than normal cells.^43,44^

## Conclusions

In this work, a novel biotin-guided piperazinyl-NBD-based fluorescent probe (NP-biotin) for sensing cellular H_2_S was successfully developed. Results confirm that NP-biotin possesses excellent sensitivity and selectivity toward H_2_S, which implies its good application in live cell imaging. The cell imaging results show that the NP-biotin could detect cellular H_2_S in complex biological systems. This work further reports that, by selecting HepG2 and LO2 as biotin receptor positive and negative cells, respectively, NP-biotin can target cancer cells *via* the recognition between the biotin group of NP-biotin and biotin receptors.

## Conflicts of interest

There are no conflicts to declare.

## Supplementary Material

RA-010-D0RA06524B-s001

## References

[cit1] Olas B. (2015). Clin. Chim. Acta.

[cit2] Wang X. B., Jin H. F., Tang C. S., Du J. B. (2010). Clin. Exp. Pharmacol. Physiol..

[cit3] Tian M., Ma Y., Lin W. (2019). Acc. Chem. Res..

[cit4] Tang Y., Ma Y., Yin J., Lin W. (2019). Chem. Soc. Rev..

[cit5] Cao D., Liu Z., Verwilst P., Koo S., Jangjili P., Kim J. S., Lin W. (2019). Chem. Rev..

[cit6] Liu X.-Q., Yan Y. (2007). Shengli Kexue Jinzhan.

[cit7] Liu Y. Y., Bian J. S. (2012). J. Alzheimer's Dis..

[cit8] Wallace J. L. (2007). Trends Pharmacol. Sci..

[cit9] Zanardo R. C. O., Brancaleone V., Distrutti E., Fiorucci S., Cirino G., Wallace J. L. (2006). FASEB J..

[cit10] Stipanuk M. H., Beck P. W. (1982). Biochem. J..

[cit11] Erickson P. F., Maxwell I. H., Su L. J., Baumann M., Glode L. M. (1990). Biochem. J..

[cit12] Qu K., Lee S. W., Bian J. S., Low C. M., Wong P. T. H. (2008). Neurochem. Int..

[cit13] Julian D., Statile J. L., Wohlgemuth S. E., Arp A. J. (2002). Comp. Biochem. Physiol., Part A: Mol. Integr. Physiol..

[cit14] Shibuya N., Tanaka M., Yoshida M., Ogasawara Y., Togawa T., Ishii K., Kimura H. (2009). Neurosci. Res..

[cit15] Hughes M. N., Centelles M. N., Moore K. P. (2009). Free Radical Biol. Med..

[cit16] Paul B. D., Snyder S. H. (2018). Biochem. Pharmacol..

[cit17] Xu Z. Q., Huang X. T., Han X., Wu D., Zhang B. B., Tan Y., Cao M. J., Liu S. H., Yin J., Yoon J. Y. (2018). Chem.

[cit18] Deng B., Ren M., Wang J.-Y., Zhou K., Lin W. (2017). Sens. Actuators, B.

[cit19] He L., Yang X., Xu K., Kong X., Lin W. (2017). Chem. Sci..

[cit20] Liu Y., Niu J., Wang W., Ma Y., Lin W. (2018). Adv. Sci..

[cit21] Lu Y., Dong B., Song W., Kong X., Mehmood A. H., Lin W. (2019). Anal. Methods.

[cit22] Wang X., Zuo Y., Zhang Y., Yang T., Lin W. (2020). Anal. Methods.

[cit23] Xu Q., He L., Wei H., Lin W. (2018). J. Fluoresc..

[cit24] Yang Y., He L., Xu K., Lin W. (2019). New J. Chem..

[cit25] Xu Z. Y., Wu Z. Y., Tan H. Y., Yan J. W., Liu X. L., Li J. Y., Xu Z. Y., Dong C. Z., Zhang L. (2018). Anal. Methods.

[cit26] Chen S., Zhao X., Chen J., Chen J., Kuznetsova L., Wong S. S., Ojima I. (2010). Bioconjugate Chem..

[cit27] Russell-Jones G., McTavish K., McEwan J., Rice J., Nowotnik D. (2004). J. Inorg. Biochem..

[cit28] Duhem N., Danhier F., Preat V. (2014). J. Controlled Release.

[cit29] Guttoff M., Saberi A. H., McClements D. J. (2015). Food Chem..

[cit30] Ozturk B., Argin S., Ozilgen M., McClements D. J. (2014). J. Food Eng..

[cit31] Vineberg J. G., Wang T., Zuniga E. S., Ojima I. (2015). J. Med. Chem..

[cit32] Guo Z., Ma Y., Liu Y., Yan C., Shi P., Tian H., Zhu W.-H. (2018). Sci. China: Chem..

[cit33] Kong X., Dong B., Zhang N., Wang C., Song X., Lin W. (2017). Talanta.

[cit34] Park S., Kim E., Kim W. Y., Kang C., Kim J. S. (2015). Chem. Commun..

[cit35] Zhang J., Zhou Y., Hu W., Lin Z., Qi H., Ma T. (2013). Sens. Actuators, B.

[cit36] Hyspler R., Ticha A., Indrova M., Zadak Z., Hysplerova L., Gasparic J., Churacek J. (2002). J. Chromatogr. B: Anal. Technol. Biomed. Life Sci..

[cit37] Olson K. R. (2009). Biochim. Biophys. Acta, Bioenerg..

[cit38] Hongfang J., Cong B., Zhao B., Zhang C., Liu X., Zhou W., Ying S., Tang C., Junbao D. (2006). Life Sci..

[cit39] Wei G., Dong R., Dong W., Lei F., Dong S., Song A., Hao J. (2013). New J. Chem..

[cit40] Brahmachari S., Ghosh M., Dutta S., Das P. K. (2014). J. Mater. Chem. B.

[cit41] Taeyoung K., Hyun Mi J., Hoa Thi L., Tae Woo K., Chulhun K., Jong Seung K. (2014). Chem. Commun..

[cit42] Li K., Qiu L., Liu Q., Lv G., Zhao X., Wang S., Lin J. (2017). J. Photochem. Photobiol., B.

[cit43] Hellmich M. R., Szabo C. (2015). Handb. Exp. Pharmacol..

[cit44] Wu D., Li M., Tian W., Wang S., Cui L., Li H., Wang H., Ji A., Li Y. (2017). Sci. Rep..

[cit45] Lee Y. H., Tang Y., Verwilst P., Lin W., Kim J. S. (2016). Chem. Commun..

[cit46] Yang J., Li K., Hou J.-T., Li L.-L., Lu C.-Y., Xie Y.-M., Wang X., Yu X.-Q. (2016). ACS Sens..

[cit47] Dong B., Song X., Kong X., Wang C., Zhang N., Lin W. (2017). J. Mater. Chem. B.

