# Supplementary material for: A biotin-guided hydrogen sulfide fluorescent probe and its application in living cell imaging
Source: RSC Adv. 2020 Oct 1;10(59):36135–40. doi: 10.1039/d0ra06524b (PMC9056979; doi:10.1039/d0ra06524b)
Supplement: RA-010-D0RA06524B-s001 [file RA-010-D0RA06524B-s001.pdf]

Electronic Supplementary Material (ESI) for RSC Advances.
This journal is © The Royal Society of Chemistry 2020

# A Biotin-Guided Hydrogen Sulfide Fluorescent Probe and Its Application in Living Cell Imaging

Chen Zhang,[‡a] Jiewen Zhang,[‡a] Zhiqiang Xu,[b] Kun Zang,[a] Feng Liu,[a] Jun Yin,[*b] Ying Tan[*a] and Yuyang Jiang[c]

[a]State Key Laboratory of Chemical Oncogenomics, Key Laboratory of Chemical Biology, Tsinghua Shenzhen International Graduate School, Tsinghua University, Shenzhen, 518055, P. R. China. Email: tan.ying@il.ccnu.edu.cn

[b]Key Laboratory of Pesticide and Chemical Biology, Ministry of Education, College of Chemistry, Central China Normal University, Wuhan 430079, China. Email: yinj@mail.ccnu.edu.cn

[c]School of Pharmaceutical Sciences, Tsinghua University, Beijing 100084, P. R. China.

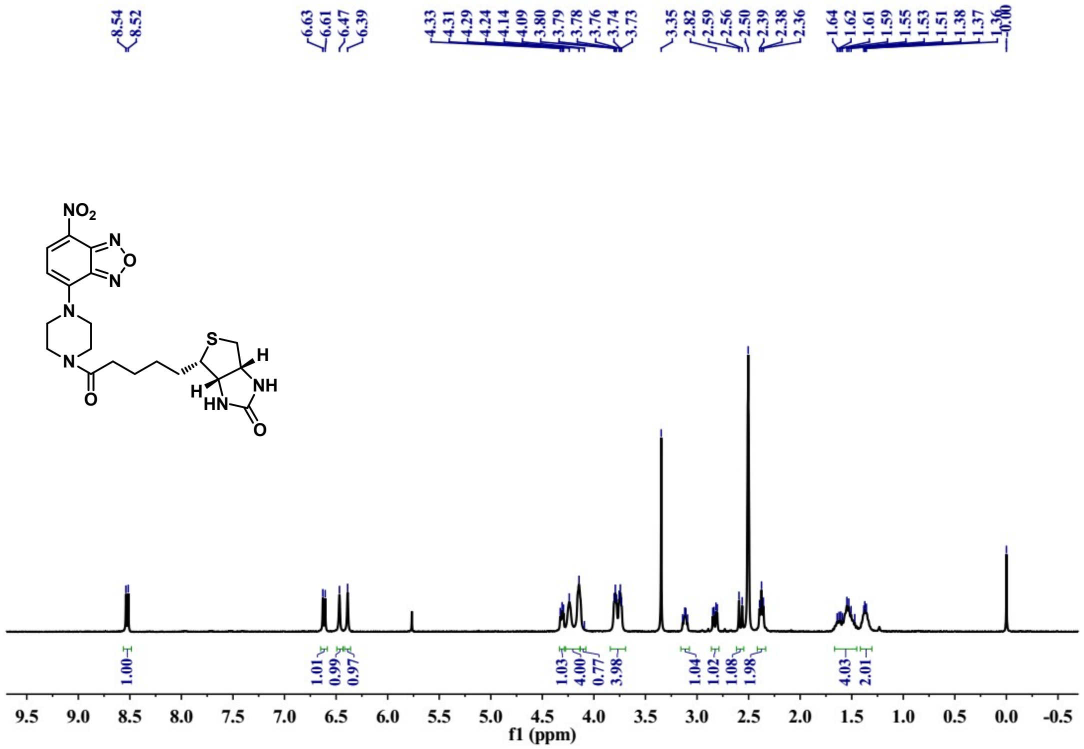


Fig. S1 $^{1}H$ NMR spectrum (DMSO-$d_6$) of NP-Biotin

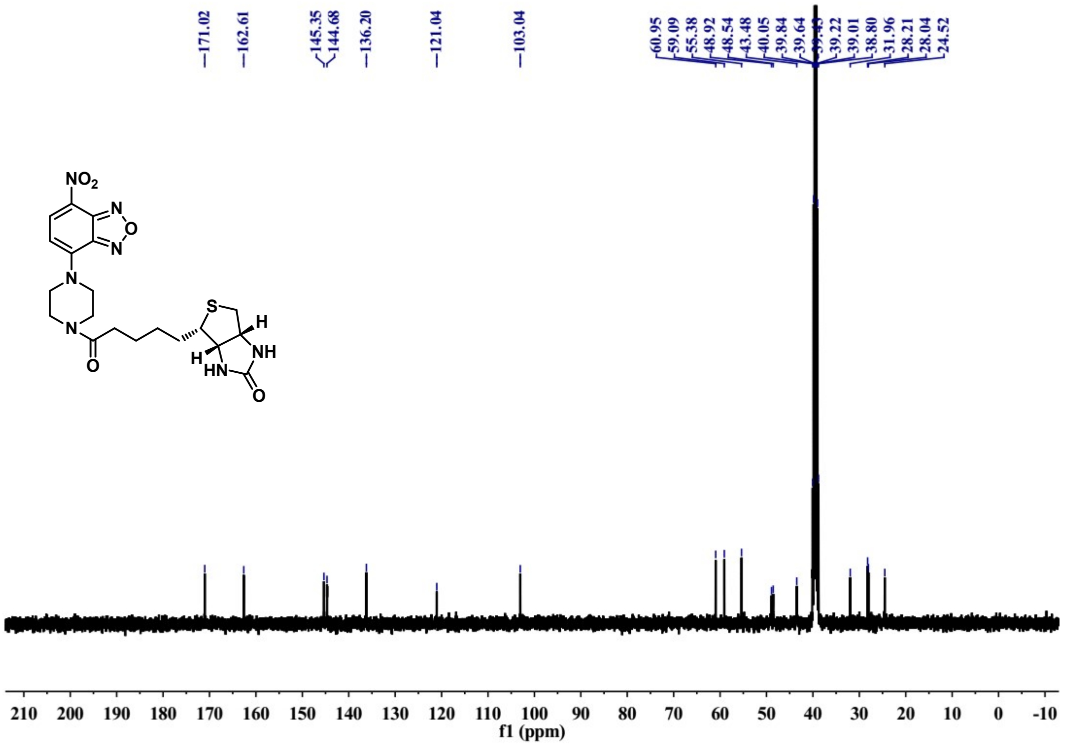


Fig. S2 $^{13}C$ NMR spectrum (DMSO-$d_6$) of NP-Biotin

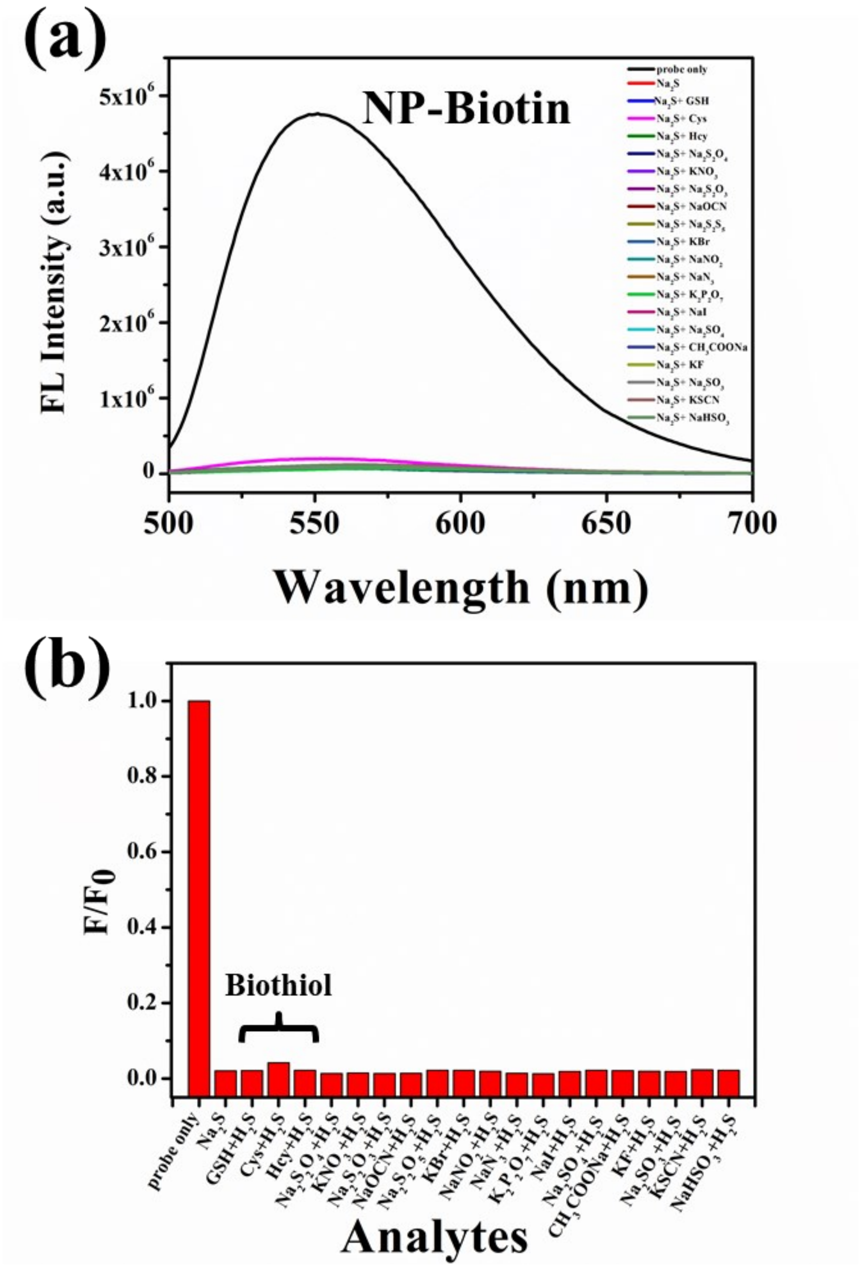


Fig. S3 Fluorescence spectra (a) and $F/F_0$ at 550 nm (b) of 10 μM NP-Biotin with $Na_2S$ in the presence of various species (GSH, Cys, Hcy, $Na_2S_2O_4$, $KNO_3$, $Na_2S_2O_3$, NaOCN, $Na_2S_2O_5$, KBr, $NaNO_2$, $NaN_3$, $K_2P_2O_7$, NaI, $Na_2SO_4$, $CH_3COONa$, KF, $Na_2SO_3$, KSCN and $NaHSO_3$) under excitation at 480 nm. ($F_0$ represents the fluorescence intensity of NP-Biotin and F represents the fluorescence intensity of NP-Biotin with $Na_2S$ in the presence of other guests respectively).

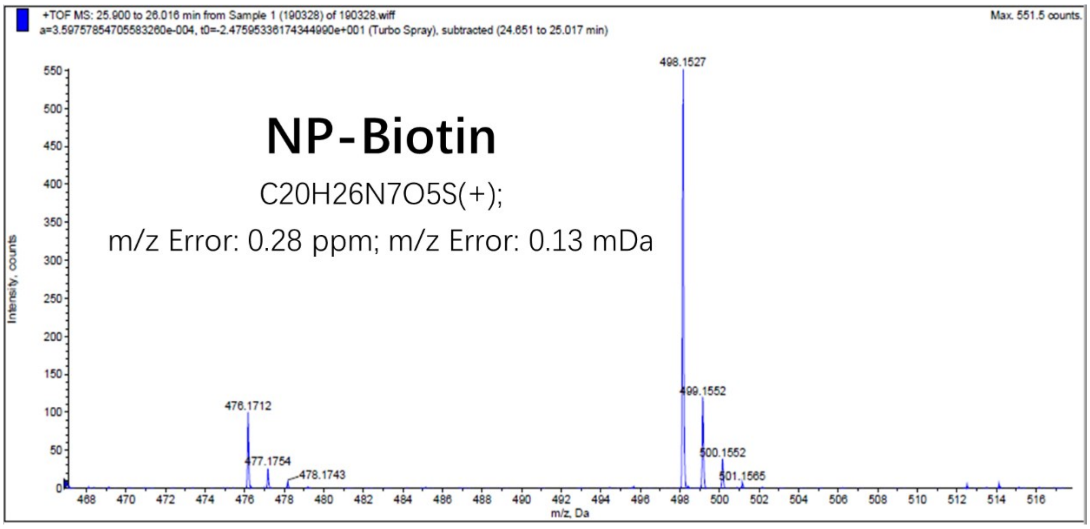


Fig. S4 HRMS study of NP-Biotin (100μM) in MeOH at room temperature

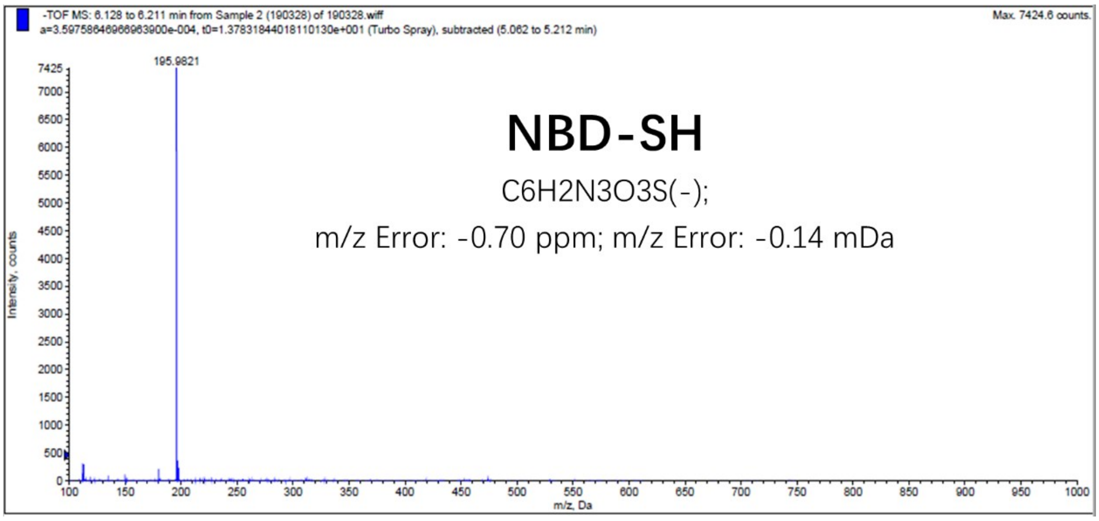


Fig. S5 HRMS study of the product of NP-Biotin (100μM) with $Na_2S$ (1 mM) in MeOH at room temperature.

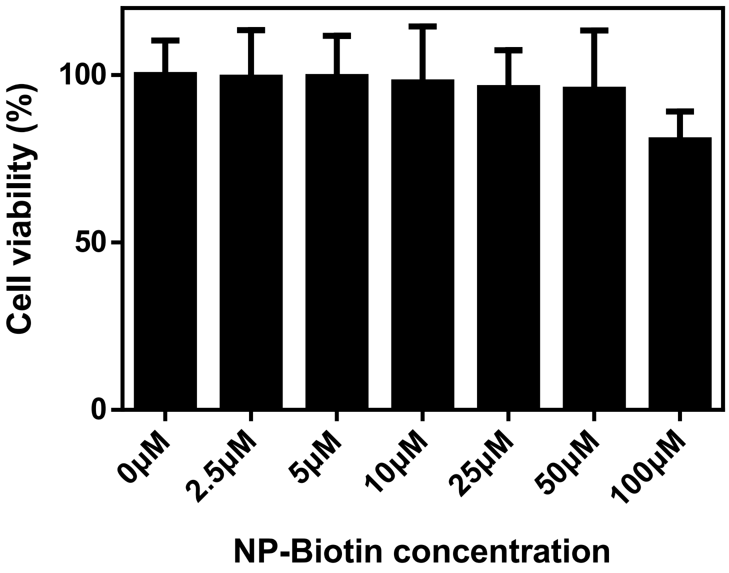


Fig. S6 Cytotoxicity of NP-Biotin towards HepG2. The cell viability was measured by CCK-8 assay.

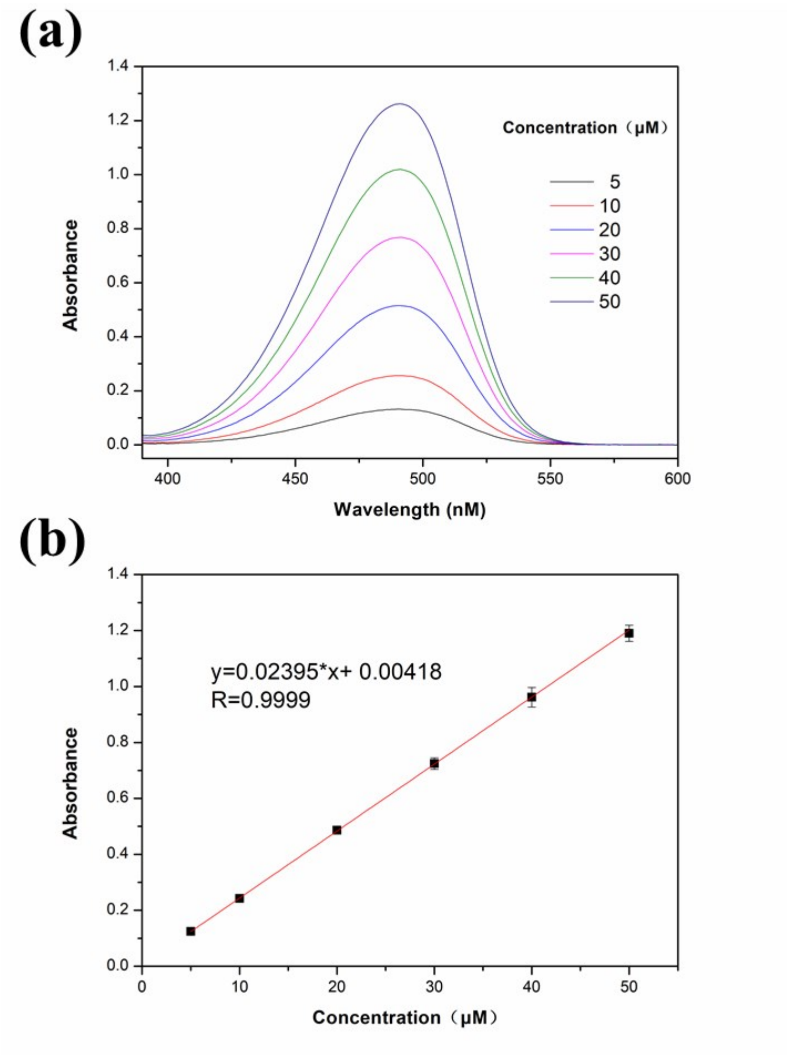


Fig. S7 (a) Fluorescence spectra of NP-Biotin at different concentrations in dimethyl sulfoxide(DMSO); (b) The linear relationship between absorbance and the NP-Biotin concentration. NP-Biotin showed maximal absorption at 490 nm ( $\varepsilon=2.395\times10^{4}L/(mol\cdot cm)$ ).

Optical properties of NP-biotin was examined in DMSO on a Cary Eclipse spectrophotometer and Fluorolog fluorescence spectrophotometer. For determination of the quantum efficiency ($\Phi_f$ ) of fluorescence, Rhodamine 6G ($\Phi$=0.95 in ethyl alcohol) was used as standards and the $\Phi_f$ value was calculated according to Equation (1)

$$\Phi_x / \Phi_{st} = [A_{st} / A_x][n_x^2 / n_{st}^2][ D_x / D_{st}] \quad (1)$$

Where st refers to the standard, x refers to the sample, A is the absorbance at the excitation wavelength, and n is the refractive index.

We calculated that the fluorescence quantum yield of NP-Biotin was 0.05 in the absence of hydrogen sulfide.

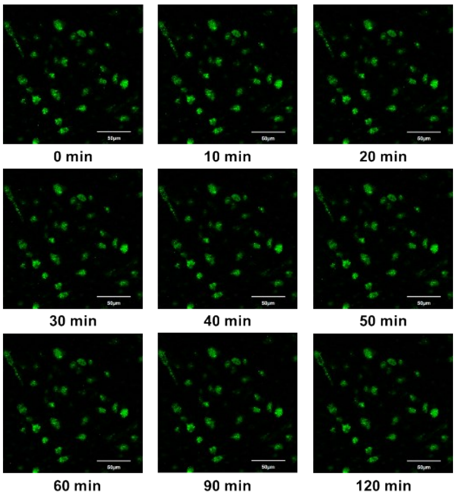


Fig. S8 Photobleaching of LO2 cells after NP-Biotin labeling. LO2 cells were imaged from 0 minute to 120 minutes after incubation with 10 μM NP-Biotin for 1 h and washing by PBS.
